# PlantCrystals—Nanosized Plant Material for Improved Bioefficacy of Medical Plants

**DOI:** 10.3390/ma13194368

**Published:** 2020-09-30

**Authors:** Abraham M. Abraham, Reem M. Alnemari, Claus Jacob, Cornelia M. Keck

**Affiliations:** 1 Department of Pharmaceutics and Biopharmaceutics, Philipps-Universität Marburg, Robert-Koch-Str. 4, 35037 Marburg, Germany; abraham.abraham@pharmazie.uni-marburg.de (A.M.A.); ph.Dreem@hotmail.com (R.M.A.); 2 Department of Bioorganic Chemistry, Universität des Saarlandes, Campus, Geb. B2.1, 66123 Saarbrücken, Germany; c.jacob@mx.uni-saarland.de

**Keywords:** nanocrystals, nanosuspension, environmental toxicity, plants, biological extracts, herbs, nanotechnology, nano-milling, high pressure homogenization, bead milling

## Abstract

PlantCrystals are obtained by milling plant material to sizes < 10 µm. Due to the disruption of the plant cells, active compounds are easily released, rendering the PlantCrystal technology an effective and low-cost process for the production of environmentally friendly plant extracts. The extracts can be used to produce phytomedicines, nutritional supplements or cosmetic products. Previous studies could already demonstrate the use of PlantCrystals to improve the antimicrobial or antifungal activity of different plants. This study investigated whether PlantCrystal technology is suitable to produce plant derived formulations with high antioxidant capacity. The study also aimed to identify the most suitable production methods for this. Methods: Various plant materials and parts of plants, i.e., seeds, leaves and flowers, and different methods were employed for the production. PlantCrystals were characterized regarding size, physical stability and antioxidant capacity (AOC). Results: PlantCrystals with particles < 1 µm were produced from the different plant materials. Both production methods, i.e., high-pressure homogenization, bead milling or the combination of both were suitable to obtain PlantCrystals. Nano milling of the plant material greatly affected their AOC and resulted in formulations with distinctly higher AOC when compared to classical extracts. Conclusions: Rendering plant material into small sized particles is highly effective to obtain plant extracts with high biological efficacy.

## 1. Introduction

Plants represent an important source for food and food products and are widely employed for the production of phytomedicines. Phytomedicines are one of the oldest pharmaceutical products and are administered as infusion (e.g., teas) or extracts. The extracts can be further modified to yield more patient convenient drug products, e.g., capsules, pellets or tablets for oral application or gels, creams and ointments for topical application. The global market for plant extracts is huge and accounted to about USD 24 billion in 2019. The compound annual growth rate (CAGR) with 16.5% is extremely high and the global market is projected to reach about USD 60 billion by 2025 [1]. The great and increasing demand in plant extracts is due to an increase in popularity of natural products and an increasing awareness of costumers regarding possible side-effects of synthetic ingredients in food, beverages or cosmetic products [1]. In addition, it is also triggered by the special health benefits offered by phytomedicines and herbal extracts when compared to chemically derived drug products [2].

The transfer of plants into plant extracts and their final products requires various steps. These can be time consuming, costly and typically require large amounts of organic solvents. In addition, they produce large quantities of organic waste, i.e., the residues of the plant material left behind after the extraction. Hence, to date, the production of plant extracts is not necessarily environmentally friendly and sustainable. Therefore, methods which improve sustainability and the environmentally friendly production of such products are of considerable importance. Issues which should be addressed in this regard are (i) an improved extraction efficacy of active compounds from the plant material, which reduces the amount of required plant material and thus saves plant material (and with this resources for cultivating, harvesting, transporting, etc. the plant material), (ii) attempts to decrease the use of organic solvents and (iii) reduction of organic waste that is created by the plant material after it has been extracted. In addition, efficient, low-cost and especially environmentally friendly processes should be developed to allow for the production of sustainable, environmentally friendly plant extracts.

**Figure 1:**
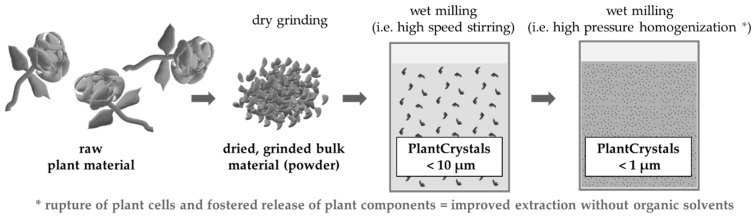
Schematic representation of the PlantCrystal principle.

In recent studies by Griffin et al. it was shown that various plant materials can be rendered into nanosized particles by using a combination of high speed stirring (HSS) and high pressure homogenization techniques (HPH) [3,4,5,6,7,8,9]. Diminution of plant materials to sizes < 10 µm means that all plant cells, which typically possess sizes > 10 µm, are destroyed. Consequently, a direct release of the active compounds from the cells without any delay is achieved (Figure 1). Thus, rendering the so called PlantCrystal technology more effective when compared to classical extraction methods where the active compounds are mostly extracted by passive diffusion through the intact walls of the plant cells.

The production of PlantCrystals is adopted from the production of drug nanocrystals. This means PlantCrystals can be simply produced by dispersing plant material powder in water to which stabilizers are added to physically stabilize the plant particles obtained as part of this process. Appropriate stabilizers comply to the regulations and requirements in the intended field of application and prevent agglomeration of the particles. They can be ionic, non-ionic or a combination of both, for example alkyl sulfates, polysorbates, phospholipids or alkyl polyglucosides. The so obtained stabilized coarse plant suspensions are subjected to HSS and subsequently HPH. The process is well established and can be run in large scale and in compliance with the GMP requirements. Hence, it is an industrially feasible and environmentally friendly process that avoids the use of organic solvents. The equipment required is low-cost, freely available and meanwhile established in many laboratories world-wide [10,11,12,13,14]. The feasibility of the PlantCrystal technology was proven in previous studies, where PlantCrystals were shown to increase the antimicrobial and antifungal activity of various plant materials [3,4,5,6,7,8,9]. Their feasibility to improve antioxidative capacity has not yet been investigated and a systematic study investigating the influence of plant material and process parameters on PlantCrystal size and properties is also not yet available.

Plants are well-recognized for their antioxidative, secondary plant metabolites and their role in preventing and treating oxidative stress and related diseases. These antioxidative properties are widely exploited in foodstuffs, beverages, functional foods, nutraceuticals, in phytomedicines and in the cosmetics field. Consequently, the demand for such extracts is large and will further increase in the coming years. Processes that allow for an efficient, sustainable and environmentally clean production of such extracts are, therefore, important to ensure not only good product quality but also a sufficient quantity without overstraining natural resources. The ability to apply the PlantCrystal technology for the production of plant extracts with high antioxidant capacity (AOC) was, therefore, investigated in this study. For this, different plant materials with well-known antioxidative properties and different parts of plants (seeds, leaves and flowers) were selected and rendered into PlantCrystals. The PlantCrystals were produced by different milling techniques and were characterized regarding their physico-chemical properties (size and zeta potential). The ability to dry and re-disperse the PlantCrystal extracts was also tested and the AOC was determined at different stages during the production.

The study was conducted in three steps. In the first step PlantCrystals were produced by using the previously applied production techniques, i.e., a combination of high speed stirring and high-pressure homogenization and subsequent freeze drying [3,4,5,6,7,8,9]. Different plant materials (leaves, seeds) with well-known antioxidative properties were selected and rendered into PlantCrystals. Their physico-chemical and biopharmaceutical properties, e.g., size, physical stability and antioxidant capacity were determined and compared to unprocessed bulk material and to the intermediate products obtained during the PlantCrystal production process. The second step of the study was performed to investigate if it is possible to produce PlantCrystals also by exploiting other methods. Therefore, in the second part of the study PlantCrystals from different plant materials (leaves, seeds, fruits) were produced by bead milling. Their physico-chemical and biopharmaceutical properties were also determined and compared to unprocessed bulk material.

**Figure 2:**
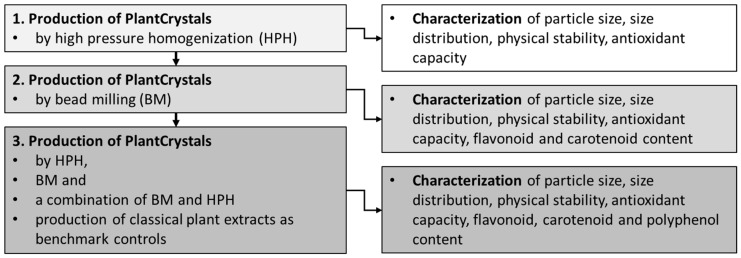
Study design, which was performed in three steps.

Based on the results obtained from the first parts, additional experiments became necessary to understand the results obtained in more detail. Therefore, the third part was performed to conduct detailed and more mechanistic experiments. The aim was to understand the influence of the production process on physico-chemical and biopharmaceutical properties of the PlantCrystals in more detail and to identify the most suitable production parameters for the production of PlantCrystals with high antioxidative capacity. Finally, the efficacy of the so obtained PlantCrystals was evaluated by comparing the AOC of the final formulations to aqueous plant extracts obtained by classical extraction methods (Figure 2).

## 2. Materials and Methods

### 2.1. Materials

For the production of PlantCrystals, plant materials with different properties were selected (Table 1). Plantacare 2000, an C8–C16 fatty alcohol polyglucoside, served as stabilizer for the formulations and was obtained from BASF AG (Ludwigshafen, Germany). Purified water was used as a dispersion medium and was obtained from a PURELAB Flex 2 (ELGA LabWater, High Wycombe, UK). All other chemicals were used as received.

### 2.2. Methods

#### 2.2.1. Production of PlantCrystals

PlantCrystals were produced by high pressure homogenization (HPH), bead milling (BM) or a combination of both methods (HPH + BM). Prior to the nano-milling process, plant materials were dry milled in an electrical grinder (Elta Lizenz GmbH, Oststeinbek, Germany and AR1105 Moulinex, Grenoble, France). The plant powders obtained were dispersed in a surfactant solution containing 1% (*w*/*w*) Plantacare 2000. All formulations were produced to contain 1% (*w*/*w*) plant material. One exception was the formulation which contained a mix of different plant materials and was based on a traditional Chinese recipe (Table 2). For the high-pressure homogenized samples, the coarse, aqueous suspension was first subjected to rotor-stator high speed stirring (HSS) to reduce the number of particles > 10 µm to a minimum (Ultra Turrax T25, IKA, Staufen, Germany, 2 min at 5000 rpm and 1 min at 8000, 10, 000 and 12,000 rpm, respectively). This pretreatment was of high importance to avoid blockage of the gap during piston-gap high pressure homogenization. HPH was performed with a LAB 40 piston gap homogenizer in discontinuous mode with a batch size of 40 mL (GEA Niro Soavi, Lübeck, Germany) in a sequence of three intervals. The first interval included three homogenization cycles at low pressure (6 × 200 bar), the second interval was a homogenization at medium pressure (3 × 500 bar, 3 × 750 bar, 6 × 1000 bar) and the third step was the high-pressure homogenization (10 × 1500 bar).

Bead milling was carried out via the small scale bead milling approach [15]. Briefly, the coarse aqueous plant suspension, Yttrium stabilized zirconium oxide beads (Ø 1 mm, SiLibeads^®^, Sigmund Lindner GmbH, Warmensteinach, Switzerland) in a ratio 60:40 and three magnetic stirring rods (size 6 mm × 10 mm) as milling shafts were placed in a small vial (12 mm × 35 mm). The vial was placed on a magnetic stirrer (IKA-Combimag RCT, IKA, Staufen, Germany) and the mixture was stirred at 1500 rpm for 24 h at room temperature. The production of the PlantCrystals with both methods was performed by subjecting the plant suspensions first to the HSS + HPH process and subsequently to the BM process, respectively.

Samples obtained were characterized directly or freeze-dried until further use. Prior to freeze drying 20% (*w*/*v*) mannitol was added to the suspensions as cryoprotectant. Freeze drying was performed by using an Alpha 1–4 LSC lyophilizer (Martin Christ Gefriertrocknungsanlagen GmbH, Osterode am Harz, Germany). The outlet temperature was set to 100 °C, and the aspirator was assigned to 100 L/min. Freeze drying was performed for 48 h at 0.1 mbar. The experiments were performed in triplicate.

#### 2.2.2. Production of Plant Extracts

Classical aqueous extracts (infusions, i.e., teas) with similar amounts of unprocessed plant material as the PlantCrystal formulations were prepared as control formulations. The extracts were prepared by placing the dry plant materials into a beaker to which heated water (80 °C) was added. After 5 min, the mixture was filtered through a metal sieve to collect the coarse plant material. Subsequently, the supernatant was filtered through filter paper with a pore size of 16 µm to collect also smaller sized plant material. The obtained aqueous extracts were cooled at room temperature and immediately used for further analysis.

#### 2.2.3. Characterization of PlantCrystals

The size of the PlantCrystals was analyzed with three independent techniques, i.e., light microscopy, dynamic light scattering (DLS) and laser diffraction (LD). The charge of the particles was estimated by analysis of the zeta potential (ZP).

Light microscopy was performed with an Olympus BX53 light microscope, which was equipped with an Olympus SC50 CMOS color camera (Olympus soft imaging solutions GmbH, Münster, Germany). DLS measurements were performed with a Zetasizer NanoZS (Malvern-Panalytical, Kassel, Germany). Measurements were conducted at 20 °C and data analyzed with the general-purpose mode built in in the software of the instrument. LD measurements were performed with a Mastersizer 3000 (Malvern-Panalytical, Kassel, Germany) and data were analyzed with Mie-theory by using 1.5 for the real and 0.01 for the imaginary index. ZP analysis was performed at 20 °C and in water adjusted to a conductivity of 50 µS/cm. The pH of the dispersion medium was in the range between 5 and 6. Measurements were performed in triplicate and the results are expressed as means ± standard deviation.

#### 2.2.4. Determination of AOC, Phenolic, Flavonoid and Carotenoid Content

The AOC expresses the strength of a given formulation to scavenge free radicals and different test methods are available for AOC analysis [18]. In this study, the AOC was determined by using the DPPH and the ORAC assay, respectively. In addition, the total phenolic content, the total flavonoid content and the total carotenoid content were determined. All measurements were performed in triplicate and the results are expressed as means ± standard deviation.

##### Determination of AOC with DPPH Assay

The DPPH assay employs 1,1-diphenyl-2-picrylhydrazyl (Sigma–Aldrich Chemie GmbH, Steinheim am Albuch, Germany) as a free radical which changes its color upon reduction by antioxidants. The changes in color can be determined photo spectroscopically. For the test, a 0.2 mM methanolic solution of DPPH was prepared freshly. A series of different concentrations of each formulation was prepared in purified water. In total, 100 µL of each obtained sample were added into the wells of a 96-well plate, mixed with 100 µL of the DPPH solution and incubated in darkness for 30 min. Afterwards, absorbance measurements were taken immediately at 517 nm (Multiskan GO, Thermo Scientific, Dreieich, Germany). Purified water served as a blank in this assay and ascorbic acid (Sigma Chemical Co., Louis, MO, USA) was the positive control [6,19,20]. The free radical scavenging activity was calculated according to the following equation:

(1) $$\%RSA=\left(A_{DPPH}-A_{sample}/A_{DPPH}\right)\times 100\%$$

In the formula *%RSA* is the radical scavenging activity, *A*_DPPH_ the DPPH absorbance and *A*_sample_ the absorbance of the sample. The sample concentrations of the sample and the corresponding *%RSA* values were plotted in a graph to obtain the corresponding IC_50_-values, i.e., the amount of formulation needed to scavenge 50% of the free radical.

##### Determination of AOC with ORAC Assay

For the ORAC (Oxygen Radical Absorbance Capacity) assay fluorescein (Alfa Aesar, ThermoFisher GmbH, Kandel, Germany) was employed as fluorescent redox indicator and AAPH (2,2′-azobis(2-methylpropionamidine) dihydrochloride, Acros Organics, Geel, Belgium) as a peroxyl radical generator. APPH has the ability to oxidize the fluorescein, thus resulting in a decay of the fluorescence intensity over time. In the presence of antioxidants, this decay in fluorescence caused by oxidants is delayed. The delay in the decay is proportional to the concentration of antioxidants being present and thus the value can be employed as a surrogate for the AOC of a formulation. The assay was performed in black opaque 96-well plates and by using different dilutions of each formulation, respectively. For each test, 20 µL of the formulation or dilution were mixed with 150 µL fluorescein solution (1 µM) and incubated for 10 min at 37 °C. Subsequently, 80 µL AAPH solution (125 mM) were added and the fluorescence intensity was measured (FluoStar^®^ Optima plate, BMG Labtech, Offenburg, Germany) every minute for 80 min at excitation and emission wavelengths of 485 and 520 nm, respectively. Trolox (6-Hydroxy-2,5,7,8-tetramethylchroman-2-carboxylic acid, Santa Cruz Biotechnology Inc., Dallas, TX, USA) solutions with concentrations in the range from 22.5 to 100 μM served as standard and ORAC values were calculated and expressed as µmol Trolox equivalents per gram of suspension [21].

##### Determination of Total Polyphenol Content

The total polyphenol content was determined by using the Folin–Ciocalteu colorimetric assay [22]. For this, 100 μL of each suspension were mixed with 200 µL of Folin–Ciocalteu’s reagent (Merck KGaA, Darmstadt, Germany) and 2 mL of purified water and incubated at room temperature for 5 min. Afterwards, 1 mL of 20% (*w*/*v*) Na_2_CO_3_ solution was added and the mix was incubated in the dark for 1 h. Finally, the reaction mixture obtained was assessed spectrophotometrically at 765 nm (Multiskan GO, Thermo Scientific, Dreieich, Germany). The content calculation was performed with respect to gallic acid (Thermo Scientific, Waltham, MA, USA) as standard, and results are expressed as gallic acid equivalent (mg GAE), respectively.

##### Determination of Flavonoid Content

The determination of the flavonoid content followed the aluminum complex formation reaction [23]. In total, 100 µL of each suspension were added along with 100 µL of 2% (*w*/*v*) AlCl_3_ ethanolic solution in a cavity of a 96 well plate and incubated in the dark for 1 h. Afterwards, the absorbance was measured at 420 nm. The flavonoid content was calculated and expressed as quercetin (Biomol GmbH, Hamburg, Germany) equivalent (µg QE/mL) with quercetin serving as a benchmark control.

##### Determination of Carotenoid Content

The carotenoid content was determined by the method of Rodriguez-Amaya et al. [24]. The absorbance of each sample was measured at 450 nm and calculations were carried out with respect to the standard curve of the beta-carotene (TCI Deutschland GmbH, Eschborn, Germany). Results are expressed as µg beta-carotene equivalent (µg β-CE/mL).

#### 2.2.5. Statistical Analysis

Analysis of the descriptive statistics and the comparison of the mean values was performed with GraphPad Prism software (version 5, GraphPad Software, Inc., La Jolla, CA, USA). Data were proven for normal distribution and variance homogeneity with the Shapiro Wilk and the Levene’s test, respectively. Multiple comparisons were subsequently performed with one-way ANOVA and post-hoc tests with Tukey correction. *p*-values < 0.05 were considered statistically significant.

## 3. Results

The aim of the study was to investigate if plant extracts with high AOC can be produced via the PlantCrystal approach. Variables that were investigated were the type of plant material, the influence of the production method and the possibility to freeze-dry the extracts for improved storability. The study was performed in three subsequent steps. The first part used high pressure homogenization (HPH) as milling principle and freeze drying as additional step to produce the PlantCrystals. In the second step bead milling was utilized of the production of the PlantCrystals and in the third step a combination of HPH and BM was employed.

### 3.1. Production and Characterization of Plantcrystals Produced by High Pressure Homogenization

**Figure 3:**
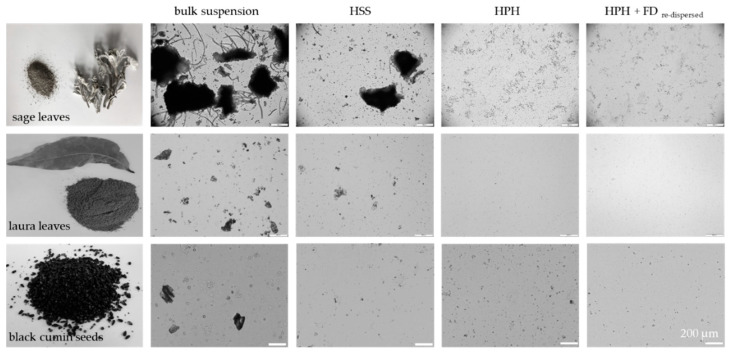
Macroscopic and microscopic images of the plant materials (sage leaves, laurel leaves and black cumin seeds) prior to and after processing with high speed stirring (HSS), high pressure homogenization (HPH) and freeze drying (FD, images taken after re-dispersion in water).

**Figure 4:**
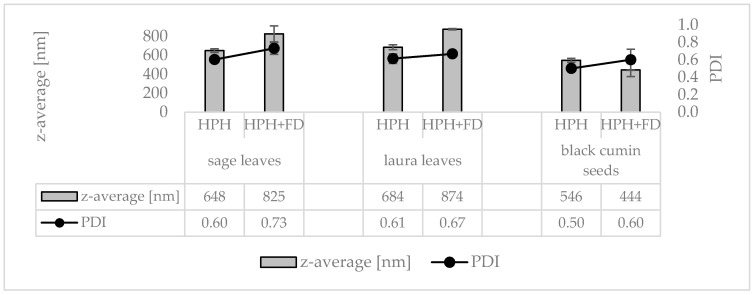
Particle sizes (DLS-data—z-average representing the mean particle size and polydispersity index (PDI) representing the broadness of the size distribution) obtained from sage leaves, laurel leaves and black cumin seeds prior to (bulk) and after high pressure homogenization (HPH) and freeze drying (HPH + FD) after re-dispersion in water.

**Figure 5:**
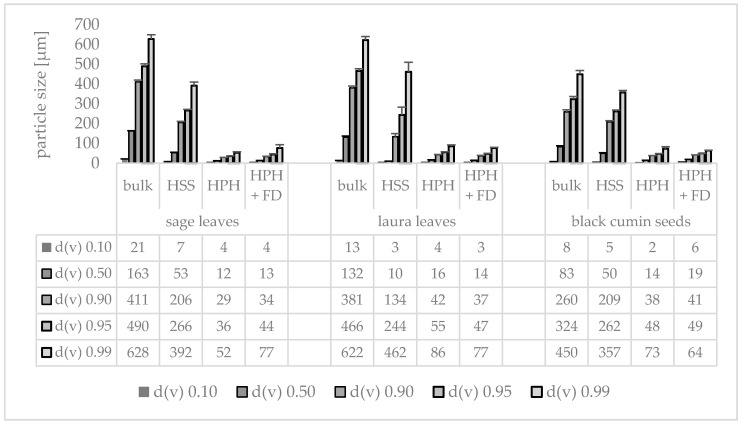
Particle sizes (LD-data) obtained from sage leaves, laurel leaves and black cumin seeds prior to (bulk) and after processing with high speed stirring (HSS), high pressure homogenization (HPH) and freeze drying (HPH + FD) after re-dispersion in water.

Rendering different plant materials (sage leaves, laurel leaves and black cumin seeds) into PlantCrystals with HSS and subsequent HPH resulted in formulations with broad size distributions, i.e., polydispersity indices >0.5. The hydrodynamic diameters were in the range between approx. 550 and 700 nm and LD data showed the presence of large quantities of microcrystals (Figure 3, Figure 4 and Figure 5). This means that HPH was able to destroy most of the plant cells which typically possessed sizes > 10 µm. Nonetheless, the transfer of the entire plant material into small-sized nanoparticles could not be achieved with this method.

Freeze drying of the PlantCrystals and re-dispersion was also possible. No changes in size were found from the microscopic images and the LD data (Figure 3 and Figure 5). DLS data revealed small changes, indicating a slight agglomeration of some individual, nanosized PlantCrystals (Figure 4).

**Figure 6:**
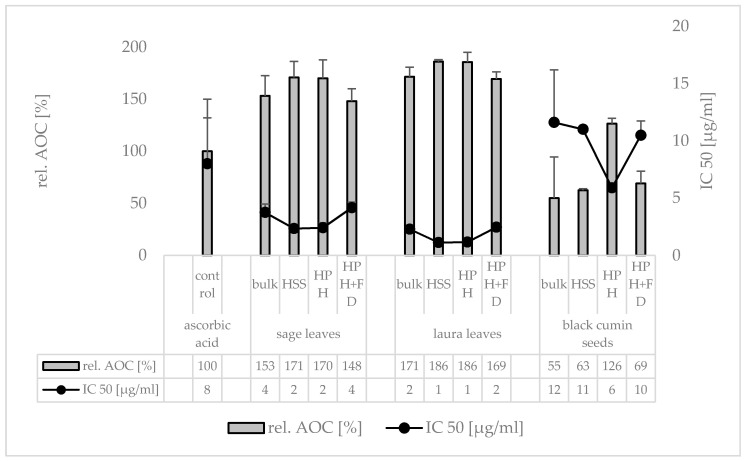
Antioxidant capacity (AOC) assessed as IC_50_ (µg/mL) from DPPH assay. The IC_50_ represents the amount of active needed to scavenge 50% of a given free radical, consequently, ow values represent high AOC. In addition, the IC_50_ values were expressed as rel. AOC (%) compared to the control.

The AOC of the plant materials was compared to the AOC of ascorbic acid. The rel. AOC represents the increase or decrease in AOC of 1 mg pure plant material when compared to 1 mg ascorbic acid (Figure 6). The bulk suspensions of the leaves possessed a higher AOC than the ascorbic acid and the black cumin seed bulk suspension possessed an AOC corresponding to about 50% of the AOC-value of the ascorbic acid. HSS increased the AOC for all plant materials by about 10% when compared to the unprocessed bulk suspension. Subsequent HPH could not further increase the AOC for the sage and laurel leaves but doubled the AUC for the black cumin leaves. This means that the forces of HSS were sufficient to destroy most of the plant cells of the leaves, but a higher energy input was required to disrupt the harder plant cells from the seeds of the black cumin. Based on the data it therefore can be concluded that HSS is sufficient for the production PlantCrystals from soft plant materials, whereas a higher energy input, i.e., HPH, is required for harder plant materials.

Freeze drying of the PlantCrystals resulted in a significant decrease in the AOC and led to AOC values being similar to the non-processed bulk suspensions (Figure 6), indicating that the freeze-drying process is possible in principle, although it would need further optimization to reduce the loss in antioxidants. Based on these findings freeze-drying was not continued in this study for the samples produced in the second and third part of the study. Instead, samples were prepared freshly and analyzed directly for their size and AOC, respectively.

### 3.2. Production and Characterization of Plantcrystals Produced by Bead Milling

In the first part of the study HPH was found to render plant material efficiently into micronized material. Unfortunately, only small quantities of nanosized PlantCrystals were obtained with this. Therefore, the second part of the study aimed at the production of small sized PlantCrystals by using another production technique. Bead milling is an established alternative technology to HPH for the production of nanocrystals and is considered to yield distinctly smaller particles when compared to HPH [25]. Therefore, to allow for a more efficient diminution of plant material to sizes < 1 µm, BM was employed in the second part of the study to render plant material into PlantCrystals.

**Figure 7:**
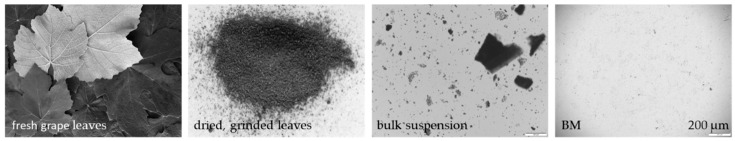
Macroscopic and microscopic images of the plant material (grape leaves fresh and dried, grinded) prior to and after processing with bead milling (BM).

The BM of grape leaves (Figure 7) resulted in finely dispersed nanosuspensions with a mean particle size below 500 nm (Table 3). The smaller particle size, when compared to HPH, was also confirmed by LD measurements which led to d(v) 0.50 values < 1 µm (Table 3). The AOC of the unprocessed grape leaves (bulk) was about 60% higher than equal amounts of ascorbic acid (Table 3). Bead milling could further increase the AOC by about 7%, although this increase was not statistically significant. The results, therefore, show that bead milling is more effective in regard to particle size diminution but seems to be not as effective in the destruction of the plant cells when compared to the HPH, which then results in a less pronounced extraction of the plant constituents and therefore in a less increased AOC.

The reduced efficacy of BM in plant cell destruction when compared to HPH is reasonable and can be explained by the different diminution principles of the different techniques. BM is a low energy, grinding process, whereas HPH is a high-energy process were particles are mainly comminuted due to cavitation. The shear forces that are created by the beads of the BM can mill the plant material to small sizes. However, it seems as though they are not able to destroy the plant cells and compartments. In contrast, HPH which is a well-known and often used process for cell lysis [26], seems to disrupt the plant cells due to the cavitational forces which occur during the homogenization process more efficiently.

Based on the results, it was assumed that a combination of HPH and BM might lead to the best results. HPH was assumed to lead to an effective rupture of the plant cells, thus leading to a powerful extraction of the antioxidants. BM was expected to comminute the remaining larger particles that were left behind from the HPH, thus leading to small sized PlantCrystals with homogenous particle size. The further decrease in size was expected to increase the physical stability of the PlantCrystals [11] and to further increase the amount of plant components extracted. Consequently, to prove this theory, the third part of the study was conducted by milling plant material with HPH, BM and with a combination of HPH and BM, respectively.

### 3.3. Production and Characterization of Plantcrystals Produced by Combined Milling Methods

**Figure 8:**
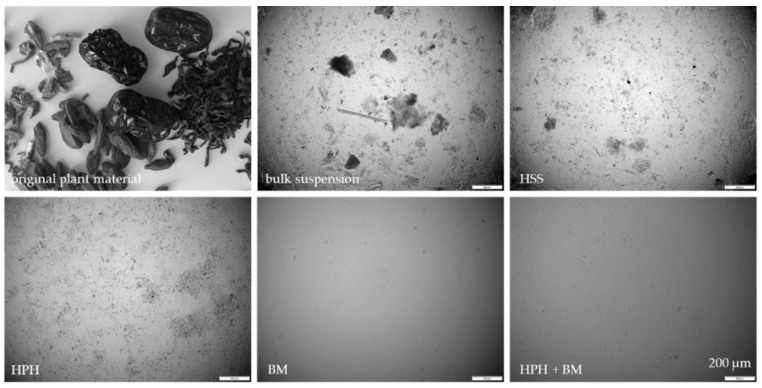
Macroscopic and microscopic images of the plant material (jasmine tea, goji berries, jujube fruits and female ginseng root) prior to and after processing with HSS, HPH, BM and the combination of HPH and BM.

**Figure 9:**
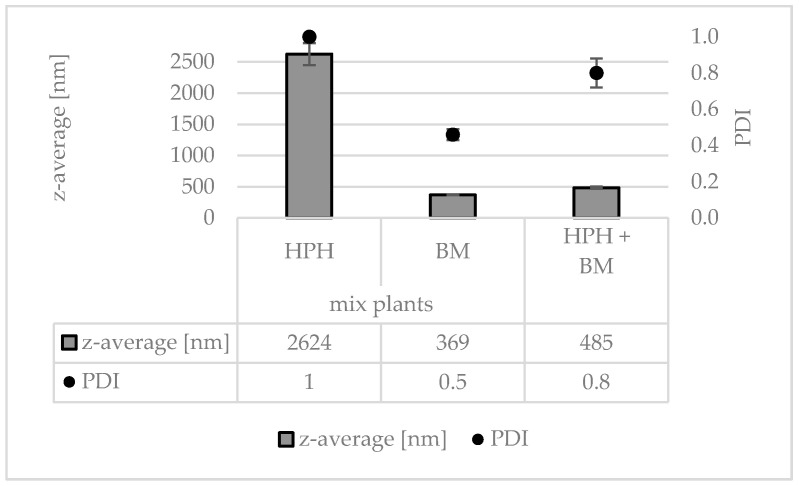
Particle sizes (DLS-data—z-average representing the mean particle size and polydispersity index (PDI) representing the broadness of the size distribution) obtained from a mix of different plant materials (jasmine tea, goji berries, jujube fruits and female ginseng root) after processing with HPH, BM and the combination of HPH and BM.

**Figure 10:**
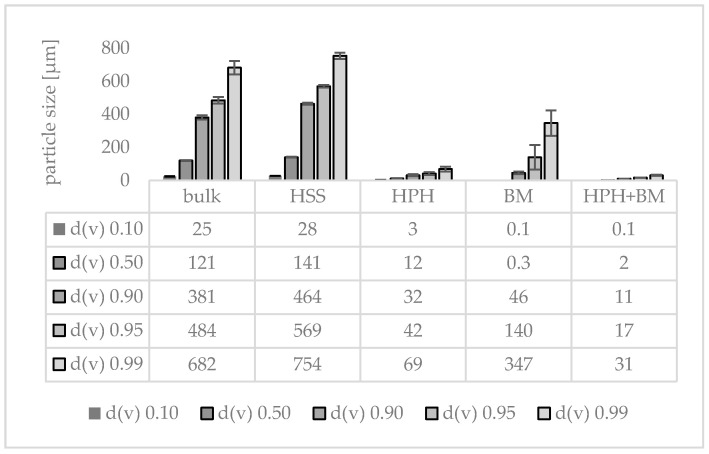
Particle sizes (LD-data) obtained from a mix of different plant materials (jasmine tea, goji berries, jujube fruits and female ginseng root) prior to and after processing with HSS, HPH, BM and the combination of HPH and BM.

Different types of plant materials (flowers, leaves, fruits, roots) were investigated in this part of the study. The plant materials (Table 2, Figure 8) were mixed, dry-grinded and subjected to HSS, HPH and subsequently BM, respectively (Figure 8, Figure 9 and Figure 10). Data confirm that BM is more effective than HPH in rendering the large sized bulk material with mean particle sizes > 100 µm into small sized PlantCrystals. DLS data show that BM resulted in PlantCrystals with sizes < 400 nm whereas HPH led to mean sizes > 2 µm (Figure 9). However, BM was less effective in the destruction of larger particles, which resulted in distinctly lager d(v) 0.95 and d(v) 0.99 values (Figure 10). The combination of HPH and BM was found to be less effective than BM alone when looking at the mean particle size (z-average, DLS data). This was not expected but might be explained by a change of the properties of the plant material during HPH. More detailed investigations are required to explain this in more detail. Nonetheless, subsequent BM after HPH was able to decrease distinctly the particle size of PlantCrystals that were obtained from the HPH alone and could also reduce the number of larger sized microparticles. This means the combination of HPH and BM resulted in a distinct decrease in the d(v) 0.9–0.99 values and the narrowest size distribution and thus seems to be the most suitable process for the production of small-sized PlantCrystals with narrow size distribution (Figure 10).

**Figure 11:**
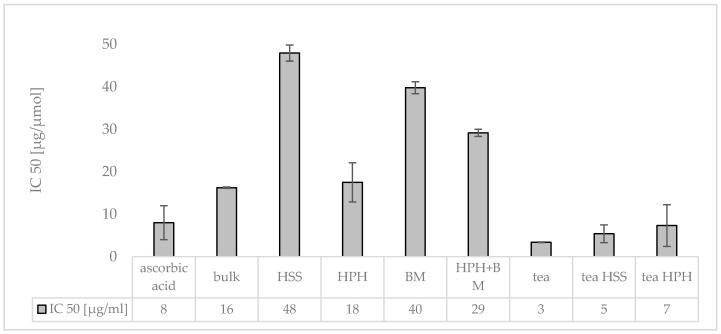
Antioxidant capacity (AOC) of PlantCrystals being composed of a mixture of jasmine tea, goji berries, jujube fruits and female ginseng root. The AOC was assessed as IC_50_ (µg/mL) from the DPPH assay. The IC_50_ represents the amount of active compound needed to scavenge 50% of the given free radical, and consequently, low values represent high AOC.

The IC_50_-values obtained from the DPPH assay for the different formulations showed that the unprocessed plant mixture (bulk) possessed about 50% of the free radical scavenging strength compared to equal amounts of ascorbic acid (Figure 11). This was determined by an approximately 2-fold higher IC_50_-value which represents the amount needed to scavenge 50% of a given amount of a free radical. After HSS the IC_50_ was 6-fold higher when compared to the control and 3-fold higher when compared to the unprocessed bulk suspension (Figure 11). The strong increase in the IC_50_-value means that HSS reduced the antioxidative potential of the bulk formulation by about 66%. BM decreased the AOC in a similar manner. Less reduction in AOC was found with HPH and with the combination of HPH and BM (Figure 11).

The decrease in AOC was not expected and the only explanation for this observation is that the plant mix contained high contents of antioxidants which are extremely sensitive to oxidation, especially in the presence of oxygen and water. In this case—due to the destruction of the plant cells—HSS would cause a pronounced release of these antioxidants from the plant material. Simultaneously, due to the high stirring speed of the rotor shaft, it would lead to a pronounced input of air (with oxygen) into the aqueous formulation, thus providing an optimal environment for an extremely fast degradation of easy oxidizable compounds.

This hypothesis is substantiated by the bead milling data (Figure 11). Bead milling also resulted in a strong decrease in AOC, although the decrease was significantly less pronounced when compared to the HSS. This is reasonable because the stirring speed during 24 h BM was only 1500 rpm but was up 12,000 rpm for the HSS. Hence, the comparatively slow stirring speed when compared to the HSS resulted in a less pronounced incorporation of air into the formulations and thus in a less pronounced decease in AOC. Nonetheless, the long milling times (24 h) with constant aeration also contributed to the degradation of the antioxidants. Another reason for the incorporation of oxygen can be attributed to the presence of the surfactant (Plantacare 2000), which easily creates foam, thus further fostering the incorporation of air into the formulations [27].

Subsequent processing of the HSS processed plant mix with HPH resulted in about a 3-fold increase in the AOC. This means that HPH was able to extract antioxidants which were not directly destroyed upon their release. The results, therefore, indicate that HPH, in contrast to HSS and BM, caused a more effective destruction of the plant cells and organelles which allowed for a more pronounced release of compounds from the plant material. Subsequent BM after HPH decreased the AOC and resulted in an increase in the IC_50_-value from 18 to 29 µg/mL (ΔIC_50_ = 11 µg/mL) (Figure 11). With this, the increase in the IC_50_-value caused by BM after HPH was only half of the increase that was caused by BM alone (ΔIC_50_ = 22 µg/mL). This means that the impact of BM on the degradation of the antioxidants was reduced significantly when HPH was performed prior to the BM.

As the BM process was kept similar, the decreased impact of BM after HPH can be attributed to the antioxidants that were released during HPH. It is reasonable that these HPH released antioxidants protected the antioxidants that were released during the subsequent BM process or vice versa. However, this explanation is not sufficient to explain the less pronounced decrease in the AOC after subsequent BM when compared to the BM alone, because the AOC of the bulk suspension was similar to the HPH processed formulation. Hence, the radical scavenging activity was similar for both formulations and thus BM should lead to a similar decrease in the AOC.

This assumption is true if one considers that BM alone and BM after HPH results in similar high extraction efficacies. However, the assumption that BM after HPH and HSS would release similar amounts of antioxidants than BM of the unprocessed bulk material is not argumentative, because large amounts of antioxidants were already released—and degraded—during the HSS process. Therefore, it is more likely that subsequent BM after HSS and HPH can extract only small quantities of antioxidants. This would then result in rather smaller AOC-values for HPH + BM formulations compared to the BM milled formulations. Due to the opposed results obtained, it was, therefore, hypothesized that the antioxidants that were released during the HPH and subsequent BM are less sensitive to oxidation than the antioxidants released during BM alone.

A possible explanation for the improved chemical stability of the antioxidants that were released during HPH and subsequent BM is the release of more lipophilic antioxidants. This is reasonable because the cavitation during HPH is known to cause a pronounced cell lysis. This could cause the rupture of cell walls and organelles which were not destroyed by BM or HSS. Cell walls and cell organelles contain various lipophilic compounds and thus it can be assumed that HPH and subsequent BM also caused the release of more lipophilic antioxidants, whereas HSS and BM cause the release of more hydrophilic antioxidants.

Lipophilic compounds are poorly soluble in water. Therefore, it is highly likely that the surfactant being present in the formulation solubilizes these compounds immediately upon their release from the plant cells into the aqueous phase. This means that the lipophilic antioxidants are localized in the hydrophobic core of the micelles and thus are more protected from oxygen than the hydrophilic antioxidants.

**Figure 12:**
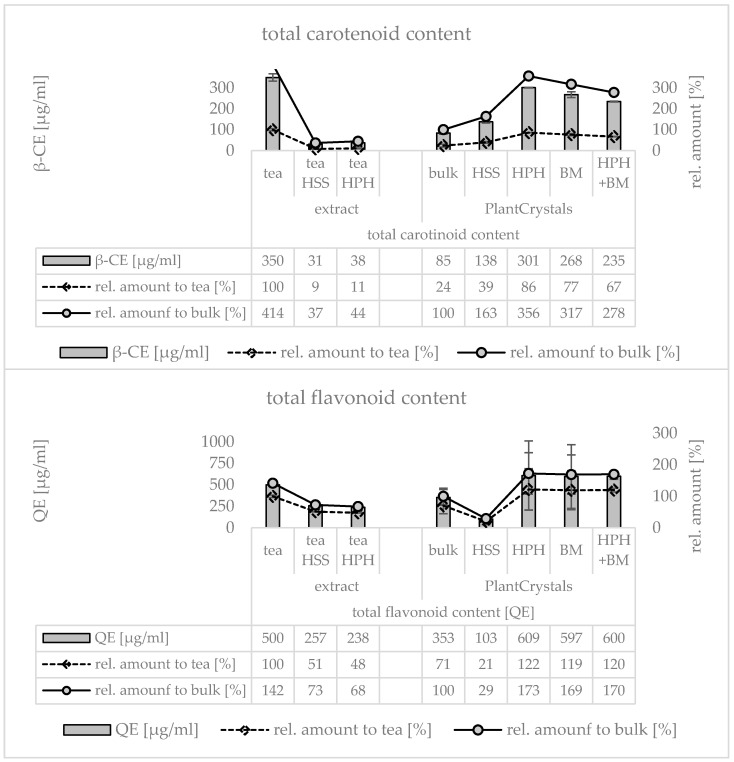
Determination of total carotenoid content (upper) and total flavonoid content (lower) of the plant mix derived tea prior to and after HSS and HPH (left) and of the PlantCrystals prior to HSS, HPH, BM and the combination of HPH and BM (right). The carotenoid content is expressed in β-carotene equivalents (β-CE) and the flavonoid content is expressed as quercetin equivalents (QE), respectively.

Data at this stage were very preliminary. Therefore, further experiments were conducted to understand the observations in more detail. First, the antioxidant capacity of an aqueous extract, i.e., tea that was prepared from the plant mix according to the traditional Chinese recipe [16,17], was determined (Figure 12 right). The AOC of this aqueous plant mix extract was about 3-fold higher when compared to equal amounts of ascorbic acid and was about 5-fold higher when compared to the unprocessed plant mix bulk suspension, thus proving that the plant mix contained high amounts of water-soluble antioxidants which were released during the preparation tea and without the need for diminution of the plant material by HSS or BM (Figure 11). Literature proves that the traditional plant mixture contains extremely high amounts of water-soluble antioxidants, mainly ascorbic acid and its derivatives derived from the goji berries and the red dates and polyphenols, e.g., epigallocatechin, from jujube and the jasmine tea [28,29].

The next step aimed at proving that HSS and HPH foster the degradation of these hydrophilic compounds. For this the freshly prepared tea was subjected to HSS and HPH, similar to the plant mixture. Subsequently, the AOC was determined. Data show an increase in the IC_50_-value to about 150% after HSS and an almost doubled IC_50_-value after HPH (Figure 12, right), thus also confirming that HSS and HPH foster the degradation of water-soluble antioxidants.

Based on the data, it was concluded that the PlantCrystal-technology is not really suitable for the extraction of chemically labile, hydrophilic compounds, because these compounds can be easily extracted without enhanced diminution of the plant material and their degradation is fostered by HSS and BM. Based on the data, it was also concluded that the PlantCrystal technology is suitable for the improved extraction of more lipophilic compounds. Therefore, the next steps aimed at investigating the influence of the PlantCrystals technology on the improved extraction of lipophilic compounds in more detail.

Lipophilic antioxidants typically derived from plant materials include, for example, vitamins, e.g., tocopherols or carotenoids, or poorly water-soluble flavonoids. Therefore, to gain more detailed information on the influence of HSS, HPH, BM and the combination of HPH and BM on the extraction efficacy of poorly water-soluble and lipophilic antioxidants, assays to determine the total flavonoid content and the total carotenoid content were performed for the PlantCrystal formulations and for the tea, respectively (Figure 12). HSS decreased the carotenoid content of the tea by about 90% and HPH caused no further reduction (Figure 12 upper). The flavonoid content of the tea was decreased by about 50% upon HSS and was not further reduced by the HPH (Figure 12 lower). The carotenoid and flavonoid contents of the PlantCrystals were also affected by the HSS and HPH. HSS caused a decrease in the flavonoid content by about 70% but increased the carotenoid content by about 60% (Figure 12). Subsequent HPH further increased the carotenoid content to about 360% compared to the unprocessed bulk suspension. The flavonoid content was increased 6-fold when compared to HSS and was about 70% higher when compared to the unprocessed bulk suspension (Figure 12 lower). No significant differences were found between HPH, BM and the formulations that were processed with the combination of HPH and BM.

The results indicate that chemical degradation occurs not only for the hydrophilic antioxidants but also for the lipophilic ones. It shows that degradation occurs mainly during HSS but not during HPH or BM, respectively. Furthermore, data also demonstrate that milling of plant material is highly effective for the extraction of flavonoids and carotenoids. Nonetheless, data could not clearly demonstrate that nano milling of plant material is superior for the extraction of flavonoids and carotenoids when compared the aqueous extraction method, i.e., the tea. There was no difference in the carotenoid content between the fresh tea and the HPH processed PlantCrystals and the flavonoid content of the HPH processed PlantCrystals was only 20% higher than that of the fresh tea (Figure 12).

A possible reason for these results is the presence of chlorophylls, which were mainly contributed from the jasmine tea leaves and resulted in a greenish color of the tea. Chlorophylls possess strong antioxidative properties and can be degraded easily upon the contact with oxygen [30]. In addition, they possess characteristic UV/vis spectra with maxima between 400 and 500 and 600 and 700 nm which might interfere with the spectra to determine the flavonoid and carotenoid contents [31]. This means it can be assumed that the values obtained for the flavonoid and the carotenoid contents represented are not only the amounts of flavonoids and carotenoids but a sum of various chlorophylls, flavonoids and carotenoids. In this case it can be assumed that the apparently high levels of carotenoids and flavonoids found in the tea are not only related to carotenoids and flavonoids but also to the chlorophylls. This would also imply that the strong decrease in the measured flavonoid and carotenoid contents measured could be attributed to the degradation of the chlorophylls and not to the degradation of the flavonoids and carotenoids only.

A simple method to investigate this assumption was the processing of plant material being rich in lipophilic antioxidants yet with low contents of chlorophylls, ascorbic acid and other fast degrading, hydrophilic antioxidants. Argan seeds serve widely as source for argan oil and thus contain high amounts of lipophilic antioxidants, e.g., tocopherols, phenols, carotenes, squalene, and unsaturated fatty acids [32]. They were, therefore, selected as a suitable candidate for the last part of this study.

**Figure 13:**
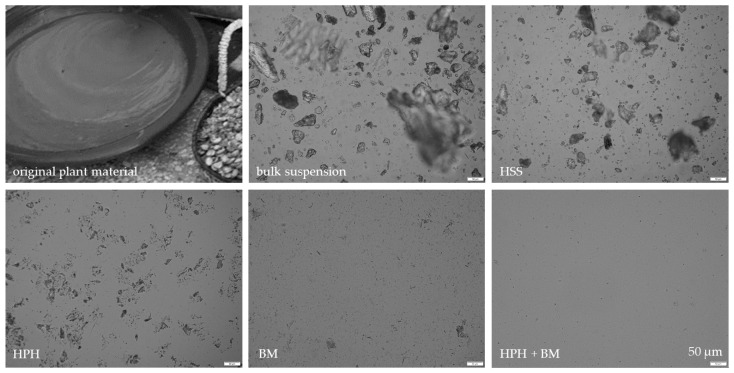
Macroscopic and microscopic images obtained from argan seeds prior to (bulk) and after processing with HSS, HPH, BM and a combination of HPH and BM.

Diminution was performed with HSS and HPH, with BM and with a combination of HPH and BM, respectively. The efficacy in diminution was comparable to the results obtained from the other plant materials, meaning that HSS with subsequent HPH was least efficient in particle diminution when compared to BM and to the combination of HPH and BM. In contrast, the combination of HPH and BM was most effective for the argan seeds and resulted in a mean particle size of about 150 nm and a very limited number of larger sized particles (Figure 13, Table 4).

**Figure 14:**
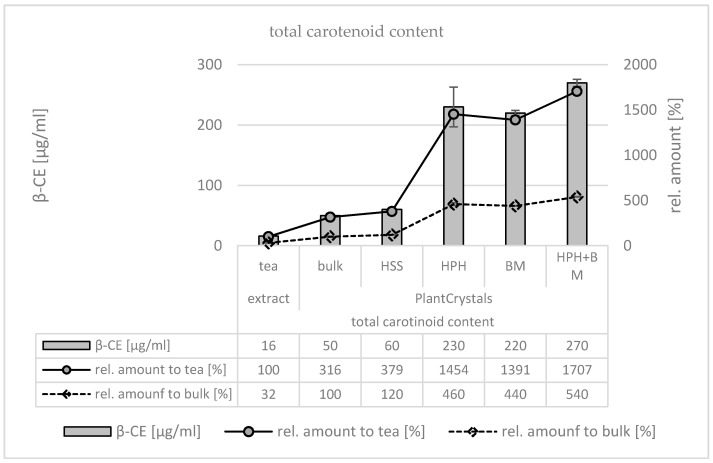

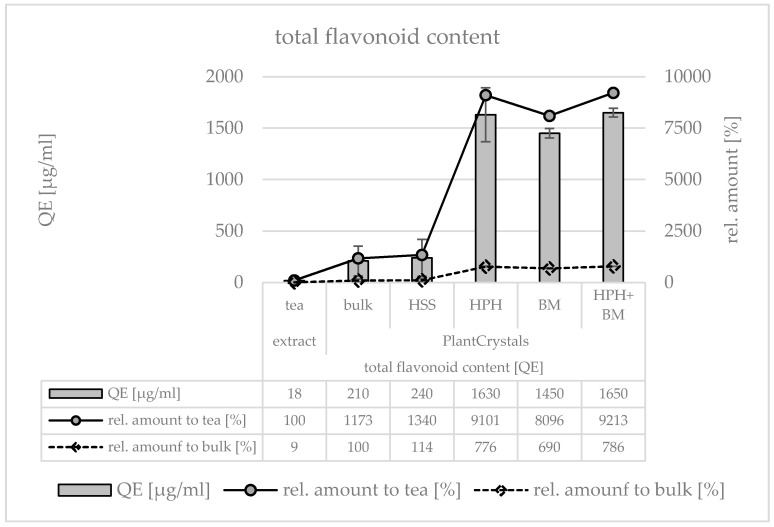
Determination of total carotenoid content (upper) and total flavonoid content (lower) of the argan seed derived tea (left) and of the PlantCrystals prior to HSS, HPH, BM and the combination of HPH and BM (right). The carotenoid content is expressed in β-carotene equivalents (β-CE) and the flavonoid content is expressed as quercetin equivalents (QE), respectively.

The determination of the flavonoid and carotenoid contents demonstrated the efficacy of the plant milling procedure (Figure 14). After HPH, BM or HPH with subsequent BM, the carotenoid content was increased about 5-fold compared to the bulk material, and the flavonoid content was about 7–8-fold higher. When compared to the tea, the increase in the carotenoid content after nano milling increased about 15-fold, and the flavonoid content was about 80–90-fold higher. HPH was slightly more effective than BM alone and the combination of HPH and BM could further increase the extraction efficacy of HPH. However, this increase was not significant. In contrast, HSS alone was not efficient and resulted in flavonoid and carotenoid contents similar to the unprocessed bulk suspension. The data, therefore, substantiate the notion that the PlantCrystal technology is especially suitable for an improved extraction of lipophilic compounds.

**Figure 15:**
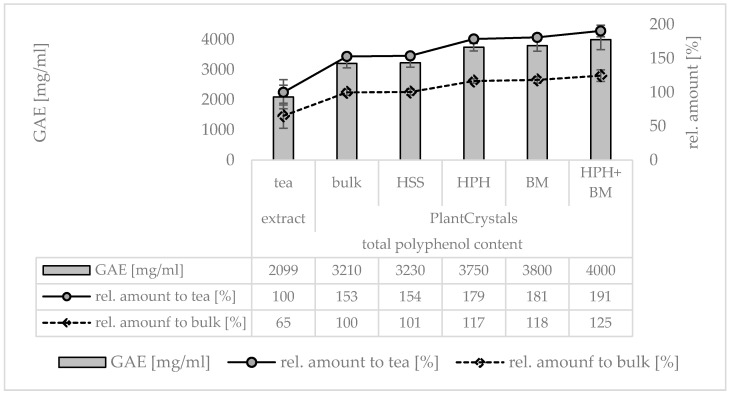
Determination of total polyphenol content of the argan seed derived tea (left) and of the PlantCrystals prior to HSS, HPH, BM and the combination of HPH and BM (right). Gallic acid was used as benchmark reference and the polyphenol content is expressed in gallic acid equivalents (GAE).

As a further proof of concept, in the next step, the formulations were analyzed regarding their total polyphenol content (Figure 15). This assay can be employed to determine polyphenols—thus including both—water soluble polyphenols, e.g., caffeic acid, ellagic, acid or gallic acid, as well as poorly water-soluble polyphenols, e.g., rutin, quercetin or resveratrol. Furthermore, other antioxidants may react and thus contribute to the values obtained. Hence, the assay is also considered to be an assay measuring the total antioxidative activity of a given formulation [33]. The results confirm the results obtained from the flavonoid and the carotenoid measurements. Hence, HSS was not effective in the extraction of antioxidants, whereas HPH, BM and the combination of these methods distinctly increased the antioxidant capacity. After nano milling, the AOC increased about 2-fold compared to the freshly prepared tea (Figure 15).

Finally, the AOC-values obtained from the Folin–Ciocalteu assay were compared to the AOC-values obtained from the DPPH assay. In the DPPH assay ascorbic acid served as a standard and reached IC_50_-values of 8 µg/mL (c.f. Table 3, Figure 6 and Figure 11). The free radical scavenging capacity of the fresh argan seed tea was about 50% of that value and the unprocessed bulk suspension had about 25% of this free radical scavenging activity (Table 5). Processing of the argan seed bulk suspension increased the AUC to up to 144% but could not reach the AOC value of the fresh tea. Hence, DPPH data, when compared to the Folin–Ciocalteu assay, were able to discriminate between differences in the AOCs between the differently processed PlantCrystal formulations but where not able to detect the distinctly higher concentration of antioxidants, i.e., carotenoids and flavonoids, in the PlantCrystal extracts when compared to the tea. A possible reason for this is the interference of the DPPH with the absorbance of carotenoids, leading to lower AOC-values of carotenoid-rich formulations [34]. Therefore, to allow for a better comparison of the AOC-values between tea and PlantCrystals, additional AOC assays are necessary to draw a more detailed picture. Various assays are available for this. From those the ORAC assay is considered to be most suitable to assess hydrophilic and lipophilic antioxidants at the same time [35]. The AOC-values obtained by the ORAC assay show a similar trend as the values obtained from the Folin–Ciocalteu assay and demonstrate an increase in AOC for the PlantCrystals upon nano milling, which finally results in a 2-fold higher AOC when compared to the AOC of the fresh tea (Table 5).

## 4. Discussion

The data obtained demonstrate that the comminution of plant material results in an accelerated release of plant components. The number of active compounds liberated was shown to be influenced by the diminution technique and by the type of plant material used. Softer materials, e.g., leaves, can be easier comminuted than harder materials, e.g., seeds. Among the production methods applied to produce the PlantCrystals high speed stirring was found to be least efficient method for the extraction of active compounds. The other methods, i.e., HPH, BM and the combination of HPH and BM revealed non-conclusive results, confirming that the extraction of antioxidants from plant materials with the PlantCrystal-technology is a rather complex process. The results indicate that the PlantCrystal extraction method is a two-step process. In the first step the plant material is milled to sizes in the upper micrometer range. This results in a pronounced release of hydrophilic antioxidants. Depending on the process and on the nature of these antioxidants, they can be degraded rather rapidly, which then results in a decrease in AOC when compared to unprocessed bulk material or freshly prepared water extracts (tea). The second step is the milling of the plant material to sizes < 1 µm. This results in an effective destruction of all plant cells and cell organelles and thus to the release of more antioxidants. The antioxidants released in the second step are more lipophilic, probably due to the destruction of the lipophilic cellular compartments that host these compounds. Consequently, smaller sizes of the PlantCrystlas can be expected to cause a higher release of these antioxidants.

In this regard BM or the combination of HPH and BM would be the most suitable production techniques for the production of PlantCrystals. These methods produced distinctly smaller and more homogenous PlantCrystals when compared to HPH. Nonetheless, the AOC-values of the final products were not higher when compared to HPH. Implying, that a small particle size cannot be the only criterium for the selection of the most suitable production method for the PlantCrystals.

The AOC of the final product represents the sum of all antioxidants released during the comminution reduced by the number of antioxidants that were degraded during the nano milling process. HSS, as performed in this study, cannot be recommended for the production of PlantCrystals, because the high stirring speed resulted in a pronounced incorporation of air. The oxygen in the air is likely to foster the oxygenation of the antioxidants. Likewise, BM for 24 h with stirring rates to up to 1500 rpm and/or a stabilizer which can promote the formation of foam were also found to cause a pronounced incorporation of air into the formulation. This resulted in oxidation of the antioxidants and thus lower AOC-values when compared to HPH. Based on the results from the study, it can be summarized that the production of PlantCrystals with high antioxidative capacity should by further improved. This means that the processes that incorporate air into the formulation should be modified. This should be possible by replacing foaming surfactants by surfactants with less foaming ability and/or by the addition of anti-foaming agents. The incorporation of oxygen can also be reduced by producing the PlantCrystals in an oxygen free environment, e.g., with an inert gas atmosphere (for example nitrogen, N_2_). The determination of the extraction efficacy can be determined by using the DPPH assay. However, for a more detailed determination of the type and amount of extracted antioxidant, other assays should also be conducted. In this way it is possible to discriminate between hydrophilic and lipophilic antioxidants and to compare the total antioxidant capacity of the PlantCrystals to their classical extracts. In this study a combination of DPPH assay, ORAC assay and assays that determine the polyphenol, flavonoid and carotenoid content were used. The combination was able to provide detailed information about the different types and amounts of antioxidants that were released during the different extraction processes. Nonetheless, the combination of these assays was not able to discriminate between chlorophylls and carotenoids and also the assay used for the determination of the polyphenol content is rather non-specific for polyphenols [36]. Therefore, for a more detailed understanding of the processes occurring during the production of PlantCrystals, it is suggested to extend the AOC test battery to at least two more tests. One test to assess the total amount of chlorophylls [37] and a second test which tests specifically for the polyphenol content of the formulation [36]. The combination of the seven tests should enable a mechanistic study to identify and improve the most relevant parameters for the production of PlantCrystals with high antioxidant capacity.

## 5. Conclusions

The PlantCrystal-technology is a simple approach for the production of environmentally friendly and sustainable plant extracts. The process requires no organic solvents and can be run on a large scale. It is thus industrially feasible and can be applied to the extraction of many plant constituents. This study showed that nano milling is especially suitable for the extraction of lipophilic and poorly water-soluble compounds. Thus, the present technology can be used to produce plant extracts with high contents of lipophilic antioxidants, which possess distinctly higher radical scavenging activities when compared to their classical, aqueous plant extracts. Future studies should improve the production parameters to reduce the amount of oxygen which is incorporated into the formulations during the nano milling process. In this way it should be possible to produce plant extracts that contain high amounts of hydrophilic and lipophilic antioxidants at the same time.

The data obtained also substantiate the findings from previous studies and demonstrate the versatile use of PlantCrystal technology for obtaining plant extracts with improved biological efficacy. Future studies should therefore utilize PlantCrystals to enhance the therapeutical potential of more medicinal plants. Besides improved antioxidative, antifungal and antibacterial activities, studies that investigate the improvement of photocatalytic properties by rendering plant materials into PlantCrystals are also suggested.

## Figures and Tables

**Table 1:** Overview of the plant materials investigated as part of this study.

Name	Binomial Name	Part of Plant	Source
sage	*Salvia officinalis* L.	leave	local market Palestine *
laurel	*Laurus nobilis* L.	leave	local market Palestine *
black cumin	*Nigella sativa* L.	seed	local market Palestine *
grape	*Vitis vinifera* L.	leave	private garden, Marburg, Germany
female ginseng	*Angelica sinensis* Diels	root	IndigoHerbs, UK
jujube-red dates	*Ziziphus jujube* Mill.	fruit	Jonnic Food Co., China
goji berries	*Lycium chinense* Mill.	fruit	BioJoy GmbH, Germany
jasmine tea	*Jasminum* L.	flower, leave	Sonnentor GmbH, Austria
argan	*Argania spinosa* L.	seed	local market Morocco **

* local market in Nablus, Palestine ** local market in Sti Fadma, Morocco.

**Table 2:** Formulation composition of plant mixture used for the PlantCrystals produced by the combination of high pressure homogenization (HPH) and bead milling (BM). The composition of the formulation is based on a traditional Chinese recipe [16,17].

Name	Latin Name	Amount
female ginseng	*Angelica sinensis*	10.0 g
jujube-red dates	*Ziziphus jujuba*	5.0 g
goji berries	*Lycium chinense* Mill.	3 pieces
jasmine tea	*Jasminum* L.	5.0 g
water	*Aqua purificata*	300 mL

**Table 3:** Particle sizes (LD-data, DLS-data) and AOC-values obtained from grape leaves prior to (bulk) and after processing with bead milling (BM).

Size & AOC Parameters		Bulk			BM		
**LD Data**	d(v) 0.10 (µm)	30	±	2	0.14	±	0.01
	d(v) 0.50 (µm)	226	±	6	0.96	±	0.6
	d(v) 0.90 (µm)	402	±	14	15	±	2.8
	d(v) 0.95 (µm)	449	±	16	26	±	4
	d(v) 0.99 (µm)	523	±	23	52	±	13
**DLS Data**	z-average (nm)	n.a.			485	±	11
	PDI	n.a.			0.5	±	0.03
	ZP (Mv)	n.a.			−19	±	0.8
**AOC**	IC_50_ (µg/mL)	3.1	±	0.7	2.1	±	0.5
	rel. AOC (%)	162	±	23	174	±	23

**Table 4:** Particle sizes (LD-data, DLS-data) and obtained from argan seeds prior to (bulk) and after processing with HSS, HPH, BM and a combination of HPH and BM.

Argan Seeds																
Size Parameters		Bulk			HSS			HPH			BM			HPH + BM		
**LD Data**	d(v) 0.10 (µm)	40	±	7	112	±	10	3	±	0	0.03	±	0	0.02	±	0
	d(v) 0.50 (µm)	199	±	4	282	±	3	12	±	7	0.10	±	0	0.07	±	0
	d(v) 0.90 (µm)	322	±	5	464	±	4	33	±	11	5	±	2	0.27	±	0
	d(v) 0.95 (µm)	355	±	5	514	±	4	43	±	17	75	±	16	128	±	14
	d(v) 0.99 (µm)	406	±	5	593	±	5	166	±	70	324	±	54	364	±	25
**DLS Data**	z-average (nm)	n.a.			n.a.			901	±	122	202	±	4	153	±	2
-	PDI	n.a.			n.a.			0.7	±	0	0.3	±	0	0.3	±	0

**Table 5:** Determination of AOC of the argan seed derived tea (left) and of the PlantCrystals prior to HSS, HPH, BM and the combination of HPH and BM (right). The AOC was determined with the DPPH assay and the ORAC assay, respectively.

AOC-Value	Tea			Bulk			HSS			HPH			BM			HPH + BM		
**IC_50_ (µg/mL)**	4	±	2	2	±	0	2	±	0	3	±	0	3	±	0	3	±	0
**Rel. AOC_DPPH_ to Bulk (%)**	171	±	58	100	±	6	93	±	6	144	±	12	105	±	4	115	±	7
**ORAC-Value (µmol/µL)**	110	±	9	118	±	31	214	±	13	204	±	8	215	±	7	179	±	3
**Rel. AOC_ORAC_ to Bulk (%)**	93	±	8	100	±	27	182	±	6	174	±	4	183	±	3	152	±	1
